# A Flexible Wireless Dielectric Sensor for Noninvasive Fluid Monitoring

**DOI:** 10.3390/s20010174

**Published:** 2019-12-27

**Authors:** Heng-Tian Zhu, Ye Chen, Yi-Feng Xiong, Fei Xu, Yan-Qing Lu

**Affiliations:** College of Engineering and Applied Sciences, Nanjing University, Nanjing 210093, China; mg1834019@smail.nju.edu.cn (H.-T.Z.); yechen@nju.edu.cn (Y.C.); dz1734004@smail.nju.edu.cn (Y.-F.X.); yqlu@nju.edu.cn (Y.-Q.L.)

**Keywords:** dielectric sensor, wireless passive, noninvasive, fluid monitoring

## Abstract

A flexible wireless dielectric sensor is presented here for noninvasively monitoring the permittivity and conductivity of fluids, based on resistor–inductor–capacitor (RLC) resonant circuit and capacitively coupled contactless conductivity detection (C^4^D) technique. The RLC sensor consists of one single-turn inductor and one interdigital capacitor. The resonant frequency of the device is sensitive to the surrounding environment, thanks to the electric field leaked out between the interdigital capacitor electrodes. Through the high-frequency structure simulator (HFSS) simulation, and experiments on ethanol/water solutions and NaCl solutions, it was confirmed that a fluid’s permittivity and conductivity could be detected by the return loss curve (S_11_). With great repeatability and stability, the proposed sensor has potential for broad applications, especially in wearable low-cost smart devices.

## 1. Introduction

In recent years, resistor–inductor–capacitor (RLC) resonators as a passive wireless sensing device are developing rapidly. RLC resonators have been widely applied for measuring temperature [1], humidity [2], strain [3], and biological analytes [4] because of their high sensitivity and long lifespan. However, the rigid properties of traditional RLC devices limit their usage in sensing fields. Thanks to the development of microfabrication technology and polymer materials, flexible RLC resonators have emerged and show great potential in specific applications such as skin hydration monitoring [5], intraocular pressure monitoring [6], intracranial pressure monitoring [7], alcohol gas detection [8], and bacteria detection [9].

With the rapid development of Internet of Things (IoT) technique, the demands for detecting the dielectric property of fluids noninvasively are growing significantly, such as pharmaceutical composition determination [10], human body fluid composition analysis [11], and food-safety testing [12]. The dielectric property of fluids can be used to reveal the information on its composition and respective concentrations. Capacitively coupled contactless conductivity detection (C^4^D) is a recently developed fluid conductivity detection technique. The C^4^D is able to detect analytes such as ions, biomolecules, and proteins [10,11,12,13,14,15,16,17] in fluids, and avoids the electrochemical reaction on electrodes. Thus, it is widely applied in capillary zone electrophoresis (CE) and high performance liquid chromatography (HPLC).

Recently, the C^4^D method combined with radiofrequency technique have attracted an increasing interest from researchers. Rogers et al. introduced a stretchable inductor–capacitor(LC)-based sensor for epidermal measurement of the volume of sweat [18]. Ma et al. introduced an LC microfluidic sensor based on Low Temperature Co-Fired Ceramic (LTCC) technology for detecting glucose with different concentrations [19]. To achieve results that are more accurate, conductivity of the fluid can be considered, which will also introduce more applications.

Here, we combine RLC resonant circuit with C^4^D technique to describe a flexible wireless dielectric sensor for noninvasively monitoring the permittivity and conductivity of fluids. The presented device consists of one single-turn inductor and one interdigital capacitor. The measured data can be easily collected by a vector network analyzer (VNA) with a readout coil through inductive coupling. The resonant frequency amplitude is used to indicate the conductivity change of the fluid to be tested. As the effect of parasitic capacitances is overcome at the resonant frequency, a higher sensitivity of conductivity is obtained [20]. In addition, the fluid’s permittivity can be detected by the drift of the resonant frequency. Thus, we realize a flexible wireless dielectric sensor that can measure the conductivity and permittivity of fluids. According to the same principle, the sensor can also detect gas. In the present study, the high-frequency structure simulator (HFSS) results, and the results from experiments with ethanol/water solutions and NaCl solutions confirm the noninvasive sensing ability of the flexible wireless dielectric sensor. With the advantages of flexibility, wireless, and noninvasive detection capability, the proposed sensor shows great potential for broad applications, such as wearable blood monitoring and real-time water quality detection.

## 2. Materials and Methods

### 2.1. Design and Operating Principle

**Figure 1:**
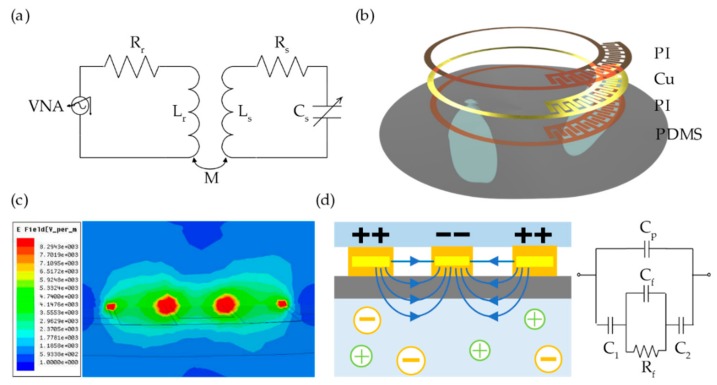
(**a**) Illustrative model schematic of resistor–inductor–capacitor (RLC) wireless measurement; (**b**) structural schematic of the proposed dielectric sensor; (**c**) electric field leaked out between the interdigital electrodes on high-frequency structure simulator (HFSS) simulation; and (**d**) schematic and equivalent circuit of the fluid monitoring.

The principle of the RLC wireless passive sensor is shown in Figure 1a. The energy and signal can be transported because the dielectric sensor and the external reading circuitry are inductively coupled. The sensor circuit, which consist of stable *L_s_*, variable *C_s_*, and variable *R_s_*, is characterized by its resonant frequency (*f_s_*) and quality factor (*Q_s_*):

(1) $$f_{s}=\frac{1}{2\pi \sqrt{L_{s}C_{s}}},\;Q_{s}=\frac{1}{R_{s}}\sqrt{\frac{L_{s}}{C_{s}}}.$$

A vector network analyzer (VNA) is used to detect the sensor’s resonant frequency through the return loss curve (*S*_11_), which is based on Equation (2):

(2) $$\frac{Z_{s}-R_{r}}{Z_{s}+R_{r}}\;Z_{s}=R_{s}+j2\pi fL_{r}\left(1+\frac{k^{2}{\left(\frac{f_{s}}{f}\right)}^{2}}{1+\frac{jf}{f_{s}Q_{s}}-{\left(\frac{f_{s}}{f}\right)}^{2}}\right),$$

where *k* is the coupling coefficient of the readout coil and sensor’s coil. When *Q_s_* ≫ 1, the S_11_ is minimal near the sensor resonant frequency because power is maximally absorbed by the resonant circuit.

The structure of the proposed dielectric sensor is shown in Figure 1b. The sensor consists of a single-turn inductor and an interdigital capacitor, which are encapsulated above and below by thin layers of polyimide (PI). The whole structure is printed to a polydimethylsiloxane (PDMS) substrate (30 µm) so that the sensor can work on an uneven surface. The planar spiral inductor has a 7.5 mm inner diameter and an 8.6 mm outer diameter, and the width of the coil is 300 µm considering the skin effect. The interdigital electrodes are approximately 700 µm long (580 µm in overlapping length) and 180 µm wide, with a uniform spacing of 30 µm between adjacent electrodes. The digits’ number of the interdigital electrodes is adjustable so that the fluid’s dielectric property in different frequencies can be discovered.

As shown in Figure 1c, the interdigital capacitor is sensitive to the dielectric property of the surrounding medium because the electric field leaks out between the interdigital electrodes. Thus, the sensor can detect the permittivity and conductivity of the fluid noninvasively, as shown in Figure 1d. In the equivalent circuit in Figure 1d, the *C_p_* represents the capacitance when the electrical field passes through the air and PDMS membrane. The *C*_1_ and *C*_2_ represent the capacitance when the electrical field passes through the tube. The fluid can be considered as a resistance parallel connection with a capacitor due to its impedance and capacitive reactance, which cannot be ignored at radio frequency. The change of the fluid’s permittivity affects the integral polarization of the surrounding medium, thus influences the capacitance of the sensor. This leads to a drift of the resonant frequency. In addition, the conductivity of the fluid interferes with the electromagnetic coupling between the sensor and the readout coil, resulting in a variation of the peak amplitude of S_11_.

### 2.2. Sensor Fabrication

**Figure 2:**
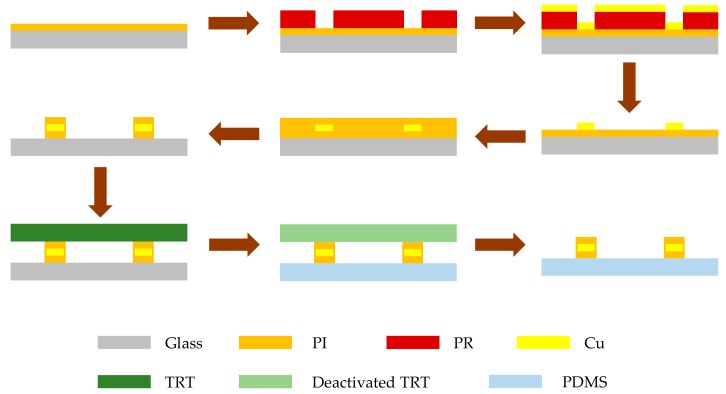
Fabrication process of the proposed RLC dielectric sensor.

In this work, we fabricated the proposed dielectric sensor using transfer printing technology, [21] which achieves a highly compatible assembly of micro/nanostructured materials on elastomers and has the advantages of excellent repeatability, simple operation, and low cost. Figure 2 illustrates the general process of fabrication.

The fabrication starts with spin coating a layer of polyimide (1 µm) onto glass at 3000 rpm. After amination at 250 °C for 40 min, a layer of photoresist (PR) is spin-coated on the PI layer and exposed to define the pattern of the inductor and interdigital capacitor. A Cu layer (2 µm) is electron-beam deposited on the PI layer using the standard lift-off technique. Then, another layer of polyimide (1 µm) is spin-coated at 3000 rpm and aminated at 120 °C for 30 min to serve as the top layer. After that, the bottom and top PI layers can be patterned using the photolithography process and etched by oxygen plasma to obtain a micro-sized pattern so that the sensor is on the glass slide. Before transfer printing, the elastomeric substrate (PDMS) is preprocessed by oxygen plasma for 2 min to clean and activate its surface. Then, the sensor can be picked up directly by the thermal release tape (TRT) and printed to the PDMS substrate by a heating step [21].

## 3. Results

### 3.1. Simulation of the Dielectric Sensor

**Figure 3:**
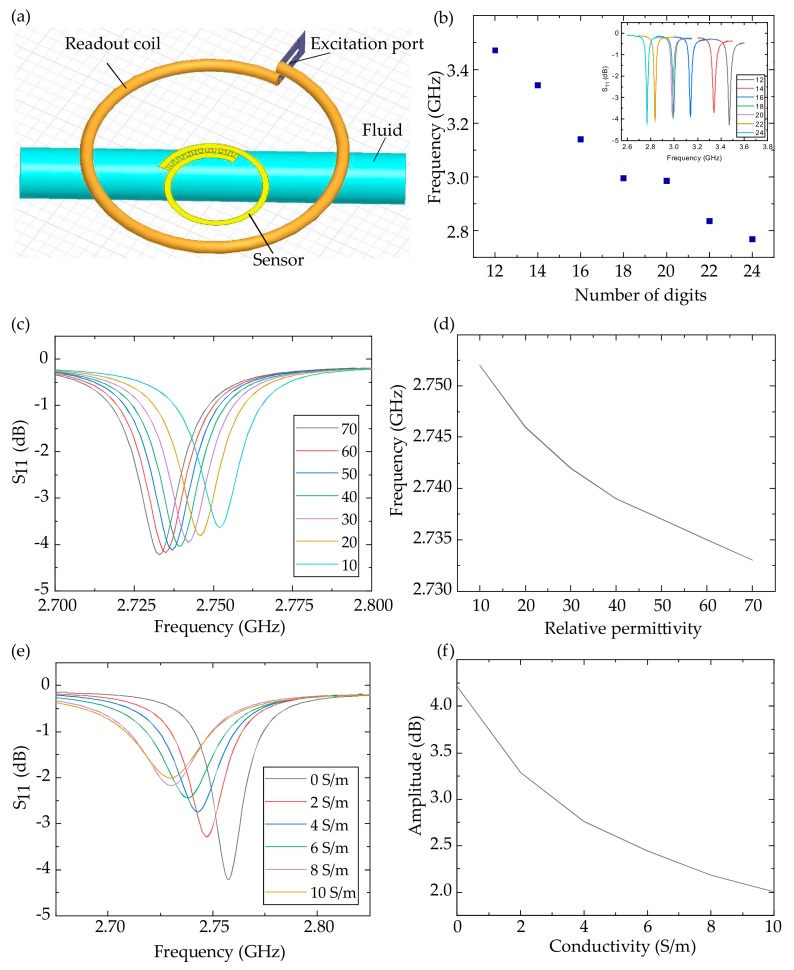
HFSS simulation of the dielectric sensor monitoring the fluid. (**a**) Model of the HFSS simulation and (**b**) the sensor’s resonant frequency with 12–24 digits. The S_11_ (**c**) and fitting results (**d**) of the fluid with different relative permittivities. The S_11_ (**e**) and fitting results (**f**) of the fluid with different conductivities.

As shown in Figure 3a, we simulated the dielectric sensor noninvasively monitoring a fluid in HFSS. The readout coil was set 3 mm above the sensor to transfer energy and detect the signal. The cylindric fluid was installed 0.3 mm below the sensor with insulating medium between them. The digits’ number of the interdigital electrodes was adjusted from 12 to 24 and the sensor’s resonant frequency was changed from 3.47 GHz to 2.77 GHz (Figure 3b). The trend of the resonant frequency drift was different when the number of digits was 20 due to the asymmetry of the structure, where the distance between the interdigital electrodes and the fluid increased a lot when the number of digits was 20. It can be concluded that by adjusting the sensor digits’ number, a fluid’s dielectric property can be detected at different frequencies so that more information is obtained for big data analytics.

More importantly, we considered the influence of the permittivity and the conductivity of the fluid respectively. We used a 24-digit sensor to detect the fluid of different relative permittivities and conductivities. As shown in Figure 3c,d, the conductivity of the fluid was set to zero and the relative permittivity was adjusted from 70 to 10. The decreased relative permittivity reduced the interdigital electrodes’ capacitance, and then increased the sensor’s resonant frequency from 2.733 to 2.752 GHz. Figure 3e,f shows that the relative permittivity of the fluid was set to 80 and the conductivity was adjusted from 0 to 10 S/m. The increased conductivity improved the medium’s polarization, so the interdigital electrodes’ capacitance was enhanced. The increased conductivity weakens the electromagnetic coupling between the readout coil and the sensor, decreasing the sensor’s resonant frequency [5] from 2.757 to 2.733 GHz and the amplitude of the S_11_ peak from 4.21 to 2.01 dB.

### 3.2. Monitoring the Dielectric Property of the Ethanol/Water Solutions

**Figure 4:**
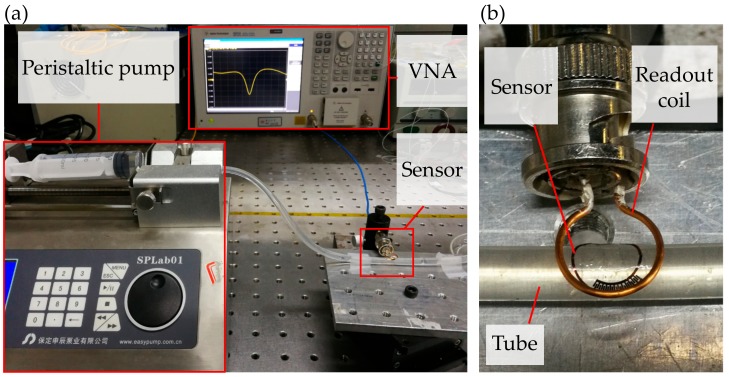
Experimental setup of the dielectric sensor monitoring the fluid. (**a**) Picture of the testing platform for the dielectric sensor. (**b**) Picture of the sensor stuck on the tube and communicated with the readout coil.

In the experiment, we fabricated a 24-digit dielectric sensor and pasted the interdigital electrode on the outside wall of a silicone tube (6 mm outer diameter and 1 mm thickness). The readout coil was 3 mm above the sensor and connected to the VNA via an external coaxial cable, as is shown in Figure 4a,b. The fluid with different dielectric property was injected into the tube using a peristaltic pump. Between adjacent tests, the previous tested liquid was pumped out and the tube was washed twice by pure water. After pumping out the pure water, the tube was blown dry to ensure the sensor’s resonant frequency recovered to the initial value. Note that all the tests were conducted at room temperature.

**Figure 5:**
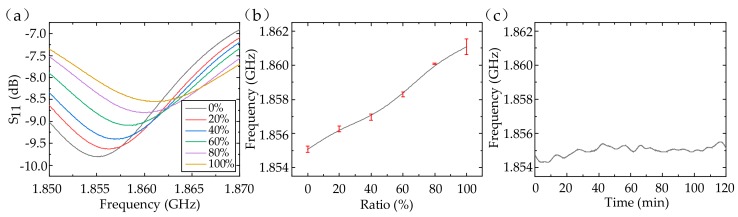
The S_11_ (**a**) and fitting results (**b**) of the ethanol/water solutions with different ethanol’s volume fraction. (**c**) Sensor’s stability with pure water injected.

First, ethanol/water solutions with ethanol’s volume fraction of 0%, 20%, 40%, 60%, 80%, and 100% were injected into the tube. The relative permittivity of the ethanol/water solution was changed from 68 to 11 [22] and the conductivity was changed from 0.90 to 1.08 S/m [23] with the ethanol’s volume fraction adjusted from 0 to 1 at the 2-GHz frequency. Because of the decrease in the permittivity, the sensor’s capacitance was reduced, resulting in an increase in the resonant frequency from 1.855 to 1.861 GHz. Therefore, we can detect the ethanol’s volume fraction in ethanol/water solution with a sensitivity of about 6.56 MHz per unit volume fraction. The average standard deviation was 0.193 MHz (about 1.04‰ in relative) among four measurements, which indicated excellent repeatability of the dielectric sensor (in Figure 5a,b). The response curve was not linear due to the existence of C_p_, C_1_, and C_2_ (in Figure 1d). Furthermore, we considered the sensor’s stability when pure water was injected (in Figure 5c). The frequency drifted between 1.85425 and 1.85563 GHz (about 3.72‰ in relative) within 2 h, showing great stability. The stability will be improved once the sensor is appropriately protected against the effect of environmental humidity.

### 3.3. Monitoring the Dielectric Property of NaCl Solutions

**Figure 6:**
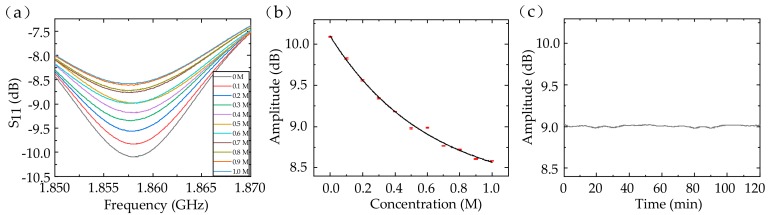
The S_11_ (**a**) and fitting results (**b**) of the NaCl solutions with different concentrations. (**c**) The sensor’s stability with 0.5 M NaCl solution injected.

The most prominent application for conductivity characterization of fluids is the ion-concentration measurement. We detected the NaCl solution’s dielectric property using a 24-digit sensor. NaCl solutions with different concentrations from 0 to 1 M were injected into the tube. The relative permittivity of the NaCl solution changed from 79 to 67 and the conductivity increased from 0 to 7.8 S/m with the concentration adjusted from 0 to 1 M at the 2-GHz frequency [24]. Because of the decrease in the electromagnetic coupling, the amplitude of the S_11_ peak declined from 10.092 to 8.582 dB. Therefore, we can detect the concentration of the NaCl solution with a sensitivity of about −1.38 dB/M. The average standard deviation was 0.00531 dB (about 5.69‰ in relative) among four measurements, which indicated excellent repeatability of the dielectric sensor (in Figure 6a,b). The resonant frequency was relatively fixed, because of the neutralization between the increase of the resonant frequency caused by the solution’s permittivity and the decrease of the resonant frequency caused by the electromagnetic coupling. Furthermore, we considered the sensor’s stability when 0.5 M NaCl solution was injected (in Figure 6c). The amplitude drifted between 8.979 and 9.025 dB (about 25.55‰ in relative) within 2 h, showing great stability.

HFSS software simulation and the experiments demonstrated that the fluid’s permittivity and conductivity mainly influenced the sensor’s capacitance and electromagnetic coupling, respectively, and then changed the S_11_. Therefore, we can noninvasively detect the concentration of a known species that affects the dielectric property of the fluid.

## 4. Conclusions

A novel flexible wireless passive dielectric sensor for noninvasive fluid monitoring was proposed in this paper. The sensor is based on RLC resonant circuitry, consisting of one single-turn inductor and one interdigital capacitor with an adjustable digits’ number. Therefore, the sensor’s resonant frequency can be regulated and more information can be obtained for big data analytics. The sensor is fabricated on glass and transferred to a PDMS substrate by thermal release tape so that it is flexible and can work on an uneven surface, such as human skin or a pipe. Through HFSS simulation and experiment, it is determined that a fluid’s permittivity influences the sensor’s capacitance, expressed as the drifted resonant frequency. Furthermore, fluid’s conductivity mostly influences the electromagnetic coupling, expressed as the drifted peak amplitude. Thus, we can noninvasively detect the concentration of a known species which affects the dielectric property of the fluid, such as the ethanol/water solution (with a sensitivity of 6.56 MHz), and NaCl solution (with a sensitivity of −1.38 dB/M). The sensitivity will increase with thinner tube’s wall. Moreover, it is feasible that the fluid’s permittivity and conductivity can be demodulated simultaneously from the return loss curve’s resonant frequency and peak amplitude. Having great repeatability and stability, the sensor shows great potential for broad applications, such as real-time blood monitoring and real-time water quality detection.
